# Validation of Syndromic Surveillance for Respiratory Pathogen Activity

**DOI:** 10.3201/eid1406.071467

**Published:** 2008-06

**Authors:** Cees van den Wijngaard, Liselotte van Asten, Wilfrid van Pelt, Nico J.D. Nagelkerke, Robert Verheij, Albert J. de Neeling, Arnold Dekkers, Marianne A.B. van der Sande, Hans van Vliet, Marion P.G. Koopmans

**Affiliations:** * National Institute for Public Health and the Environment, Bilthoven, the Netherlands; † United Arab Emirates University, Al-Ain, United Arab Emirates; ‡ Netherlands Institute of Health Services Research, Utrecht, the Netherlands

**Keywords:** respiratory tract infections, epidemiology syndrome, population surveillance, disease outbreaks, humans, research

## Abstract

Syndromic surveillance is increasingly used to signal unusual illness events. To validate data-source selection, we retrospectively investigated the extent to which 6 respiratory syndromes (based on different medical registries) reflected respiratory pathogen activity. These syndromes showed higher levels in winter, which corresponded with higher laboratory counts of *Streptococcus pneumoniae*, respiratory syncytial virus, and influenza virus. Multiple linear regression models indicated that most syndrome variations (up to 86%) can be explained by counts of respiratory pathogens. Absenteeism and pharmacy syndromes might reflect nonrespiratory conditions as well. We also observed systematic syndrome elevations in the fall, which were unexplained by pathogen counts but likely reflected rhinovirus activity. Earliest syndrome elevations were observed in absenteeism data, followed by hospital data (+1 week), pharmacy/general practitioner consultations (+2 weeks), and deaths/laboratory submissions (test requests) (+3 weeks). We conclude that these syndromes can be used for respiratory syndromic surveillance, since they reflect patterns in respiratory pathogen activity.

## Abstract 2

The studied respiratory syndromes are suitable for syndromic surveillance because they reflect respiratory pathogen activity patterns

Early warning surveillance for emerging infectious disease has become a priority in public health policy since the anthrax attacks in 2001, the epidemic of severe acute respiratory syndrome in 2003, and the renewed attention on possible influenza pandemics. As a result, new surveillance systems for earlier detection of emerging infectious diseases have been implemented. These systems, often labeled “syndromic surveillance,” benefit from the increasing timeliness, scope, and diversity of health-related registries (*1*–*6*). Such alternative surveillance uses symptoms or clinical diagnoses such as “shortness of breath” or “pneumonia” as early indicators for infectious disease. This approach not only allows clinical syndromes to be monitored before laboratory diagnoses, but also allows disease to be detected for which no additional diagnostics were requested or available (including activity of emerging pathogens). Our study assessed the suitability of different types of healthcare data for syndromic surveillance of respiratory disease.

We assumed that syndrome data—to be suitable for early detection of an emerging respiratory disease—should reflect patterns in common respiratory infectious diseases (*7*–*10*). Therefore, we investigated the extent to which time-series of respiratory pathogens (counts per week in existing laboratory registries) were reflected in respiratory syndrome time-series as recorded in 6 medical registries in the Netherlands. We also investigated syndrome variations that could not be explained by pathogen counts. As an indication for syndrome timeliness, we investigated the delays between the syndrome and pathogen time-series.

## Methods

### Syndrome Data Collection and Case Definitions

We defined syndrome data as data in health-related registries that reflect infectious disease activity without identifying causative pathogen(s) or focusing on pathogen-specific symptoms (such as routine surveillance data for influenza-like illness [*11*] or surveillance of acute flaccid paralysis for polio [*12*]).

Registries for syndrome data were included if they met the following criteria: 1) registration on a daily basis;2) availability of postal code, age, and sex; 3) availability of retrospective data (>2 years); and 4) (potential) real-time data availability.

Six registries were selected (Table 1) that collected data on work absenteeism, general practice (GP) consultations, prescription medications dispensed by pharmacies, diagnostic test requests (laboratory submissions) (*13*), hospital diagnoses, and deaths. In all registries, data were available for all or a substantial part of 1999–2004. For the GP, hospital, and mortality registry, definition of a general respiratory syndrome was guided by the case definitions and codes found in the International Classification of Diseases, 9th revision, Clinical Modification (ICD-9-CM), as selected by the Centers for Disease Control and Prevention (Atlanta, GA, USA) (www.bt.cdc.gov/surveillance/syndromedef). For the laboratory submissions and the pharmacy syndrome, we selected all data that experts considered indicative of respiratory infectious disease (for detailed syndrome definitions, see Technical Appendix).

**Table 1:** Registries from which syndrome data were obtained, the Netherlands, 1999–2004*

Data type	Period	% Coverage†	Respiratory syndrome definitions‡	Analyzed data	International code system	Registry
Absenteeism	2002–2003	80§	Reported sick employees; no further medical information	Sick leave reports of employees	–	Statistics Netherlands (CBS), www.cbs.nl
General practice consultations	2001–2004	1–2	Symptoms and diagnoses indicating respiratory infectious disease	Symptoms and diagnoses recorded in practice or telephone consultations and in home visits	ICPC	Netherlands Information Network of General Practice (LINH), www.nivel.nl/linh
Pharmacy dispensations	2001–2003	85	Prescribed medications indicative for respiratory infectious disease	Prescription medications dispensed in Dutch pharmacies, coded according to the WHO ATC classification	ATC	Foundation for Pharmaceutical Statistics, http://www.sfk.nl
Hospitalization	1999–2004	99	General respiratory symptoms/diagnoses; specific respiratory biologic agent diagnoses	Discharge and secondary diagnoses, date of hospitalization	ICD-9-CM	Dutch National Medical Register (LMR)
Laboratory submissions¶	2001–2004 (1999–2000 excluded due to unstable coverage)	16	All submissions for microbiologic diagnostic tests on respiratory materials; all submissions for serologic testing on known specific respiratory pathogens; all submissions for *Legionella* or *Streptococcus pneumoniae* antigen tests on urine	Laboratory submission requests for diagnostic testing	–	National Infectious Diseases Information System (ISIS) (*13*)
Mortality	1999–2004	100	General respiratory symptoms/diagnoses; specific respiratory biologic agent diagnoses	Date of death, primary cause of death, complicating factors, other additional causes of death	ICD-10	CBS

*ICPC, International Classification of Primary Care; WHO, World Health Organization; ATC, Anatomic Therapeutic Chemical Classification System; ICD-9-CM, International Classification of Diseases, 9th revision, Clinical Modification; ICD-10, International Classification of Diseases, 10th revision. †Percentage of total population, 16.3 million. ‡For detailed syndrome definitions and codes, see Technical Appendix.  §Percentage of working population, 8 million. ¶Diagnostic test requests with both negative and positive results.

### Respiratory Pathogen Counts

As a reference for the syndrome data, we included specific pathogen counts for 1999–2004 from the following sources: 1) Weekly Sentinel Surveillance System of the Dutch Working Group on Clinical Virology (which covers 38%–73% of the population of the Netherlands [*14*] for routine laboratory surveillance of respiratory syncytial virus [RSV], influenza A virus, influenza B virus, rhinovirus, *Mycoplasma pneumoniae*, parainfluenza virus, enterovirus, and adenovirus); 2) 6 regional public health laboratories for respiratory disease–related counts of *Streptococcus pneumoniae* (data in 2003–2004 were interpolated for 2 laboratories during short periods of missing data; total coverage 24%); and 3)national mandatory notifications of pertussis. The networks for respiratory pathogen counts are other networks than the earlier described laboratory submissions network for syndrome data.

### Data Analysis and Descriptive Statistics

Data were aggregated by week and analyzed by using SAS version 9.1 (SAS Institute Inc., Cary, NC, USA). For the GP, pharmacy, and laboratory submissions registries, we expressed the respiratory counts as a percentage of total weekly counts to adjust for the influence of holidays and, for laboratory submissions, changes in the number of included laboratories over time. By looking at the graphs, we explored the relationship between the time-series of respiratory pathogens and syndromes and calculated Pearson correlation coefficients.

### Linear Regression Models

To investigate whether the respiratory syndromes reflect patterns in respiratory pathogen counts, we constructed multiple linear regression models. These models estimated respiratory syndrome levels at a certain time with, as explanatory variables, the lagged (range of –5 to +5 weeks) pathogen counts as explanatory variables. We used linear regression of the untransformed syndrome to estimate the additive contributions of individual pathogens to the total estimated syndrome. We assumed a constant syndrome level attributable to factors other than the respiratory pathogens and constant scaling factors for each of the lagged pathogens. A forward stepwise regression approach was used, each step selecting the lagged pathogen that contributed most to Akaike’s information criterion of model fit (*15*). Each pathogen entered the model only once and only if it contributed significantly (p<0.05). Negative associations (e.g., between enteroviruses, which peak in summer, vs. respiratory syndromes, which peak in winter) were excluded to avoid noncausal effects.

To discriminate between primary and secondary infections by *S. pneumoniae* (as a complication of respiratory virus infection) (*16*–*19*), we used the residuals from regressing *S. pneumoniae* counts on other pathogens as the variable for *S. pneumoniae* (instead of its counts) for all the earlier described models for respiratory syndromes.

We checked for autocorrelation in the residuals of the models with hierarchical time-series models (using SPLUS 6.2) (*20*,*21*). We calculated R^2^ values to estimate to what extent respiratory pathogen counts explain variations in syndromes. To explore to what extent seasonal variation could be a confounder, we also calculated R^2^ values of the models after adding seasonal variables (sine and cosine terms) and R^2^ values for seasonal terms alone. We also investigated the pathogen-specific effects in the models, by calculating the standardized parameter estimates before and after adding seasonal terms.

The models were used to estimate the expected syndrome level with 95% upper confidence limits (UCLs). We considered distinct syndrome elevations that exceeded the UCLs, as unexplained by the models (for model details, see Technical Appendix).

### Timeliness

We investigated the timeliness of the registry syndromes in 2 ways: 1) as a measure of differences in timeliness between registries, we evaluated the time delays of the syndromes relative to each other by calculating for each of the syndromes the time lag that maximized Pearson correlation coefficient with the hospital registry (as a reference); 2) by estimating the time delays between each of the syndromes and the lagged pathogens included in its regression model.

## Results

### Data Exploration and Descriptive Statistics

**Figure 1:**
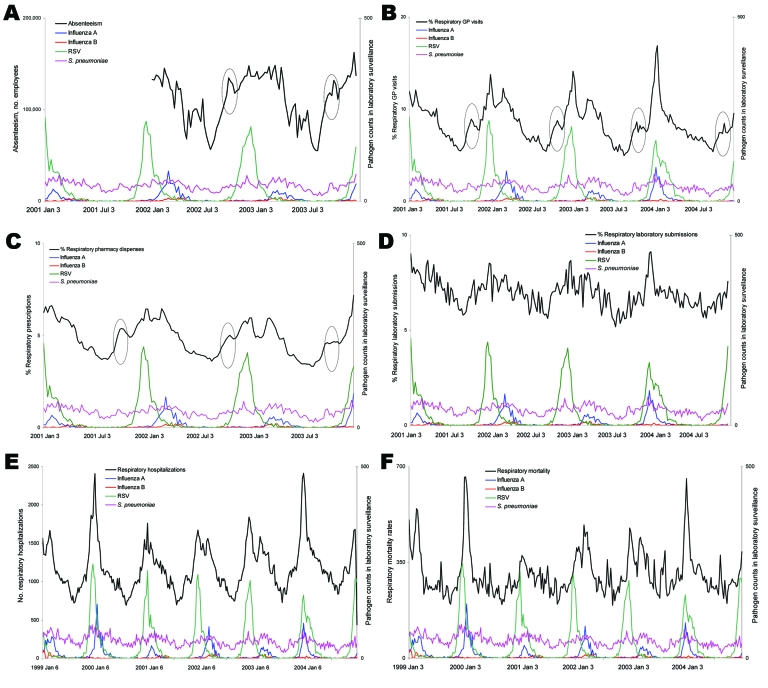
Respiratory syndrome time series and laboratory pathogen counts in the Netherlands. Respiratory syndromes were defined for the 6 registries defined in Table 1: A) absenteeism, B) general practice (GP) consultations, C) pharmacy, D) laboratory submissions, E) hospitalizations, and F) mortality counts. Pathogens plotted were respiratory syncytial virus (RSV), influenza A, influenza B, and *Streptococcus pneumoniae* [1999–2004 or part of this period, panels A–C]. Recurrent unexplained syndrome elevations in October are circled. Pathogen counts are daily counts of pathogens found in laboratory survellience.

Respiratory syndrome time series were plotted for all registries (Figure 1). The Christmas and New Year holidays coincided with peaks and dips in the pharmacy and absenteeism syndromes (not shown). Because these results were probably artifacts, we smoothed these yearly peaks and dips and censored them in the analyses performed on the absenteeism registry, in which they had a strong influence on outcomes. For all registries, the respiratory syndromes demonstrated higher levels of activity in winter, which overlapped or coincided roughly with the seasonal peaks of influenza A, influenza B, RSV, and (albeit less pronounced) *S. pneumoniae* laboratory counts (Figure 1). Infections with parainfluenza virus, *M. pneumoniae*, adenovirus, and rhinovirus were detected slightly more frequently during winter (data not shown). *Bordetella pertussis* and enterovirus showed seasonal peaks only in summer (data not shown).

The seasonal peaks in laboratory counts of influenza A, influenza B, and RSV corresponded with peaks in the GP, pharmacy, and hospital syndromes. Other syndromes did have less obvious correspondence. Each year, around October, the respiratory syndrome showed a peak in the GP (2001–2004), pharmacy (2001–2003), and absenteeism (2002–2003) registries (Figure 1, **panels A**–**C**) that was observed neither for the other registries nor in any of the laboratory pathogens.

We calculated Pearson correlation coefficients between the different unlagged time series of respiratory pathogens and syndromes (Table 2). Syndrome time series in all registries correlated strongly with *S. pneumoniae* (unadjusted total counts). The hospital, GP, pharmacy, and laboratory submissions data strongly correlated with RSV and influenza A counts (Table 2). Mortality data correlated strongly with influenza A (r = 0.65) and influenza B (r = 0.50) infections. The highest correlations between pathogen time series were between *S. pneumoniae* and the other pathogens (up to 0.51 with influenza A, Table 3).

**Table 2:** Pearson correlation coefficients between time series of syndromes and laboratory pathogen counts, the Netherlands, 1999–2004*†

Pathogen	Hospital	GP	Mortality	Pharmacy	Laboratory submissions	Absenteeism
RSV	**0.74**	**0.67**	0.41	**0.58**	**0.53**	0.47
Influenza A	**0.57**	**0.61**	**0.65**	**0.60**	0.47	0.35
Influenza B	0.31	0.39	**0.50**	0.42	0.34	0.33
*Streptococcus pneumoniae*	**0.73**	**0.71**	**0.56**	**0.75**	**0.58**	**0.69**
Rhinovirus	0.33	0.34	0.33	0.33	NS	0.35
Parainfluenza	0.20	NS	NS	NS	0.25	NS
Adenovirus	0.37	0.35	0.33	0.36	NS	0.34
Enterovirus	−0.65	−0.66	−0.59	−0.61	−0.57	−0.51
*Mycoplasma pneumoniae*	0.13	0.27	0.25	0.39	0.32	0.26
*Bordetella pertussis*	NS	NS	NS	NS	NS	NS

*GP, general practice; RSV, respiratory syncytial virus; NS, nonsignificant. Correlations >0.50 in **boldface**; p>0.05. †Unlagged.

**Table 3:** Pearson correlation coefficients between time series in respiratory pathogen counts, the Netherlands, 1999–2004*†

Pathogen	*S. pneumoniae*	RSV	Influenza A	Influenza B	RV	PIV	Adenovirus	Enterovirus	*Mycoplasma pneumoniae*	*Bordetella pertussis*
*S. pneumoniae*	1.00	0.35	**0.51**	0.36	NS	0.32	0.32	–0.44	0.21	−0.31
RSV		1.00	0.23	NS	0.30	0.13	0.21	–0.30	0.19	NS
Influenza A			1.00	0.36	NS	0.12	0.24	–0.39	0.16	−0.25
Influenza B				1.00	NS	NS	NS	–0.30	0.25	−0.21
RV					1.00	NS	0.21	NS	NS	Ns
PIV						1.00	NS	–0.19	NS	NS
Adenovirus							1.00	–0.21	NS	−0.14
Enterovirus								1.00	−0.15	0.21
*M. pneumoniae*									1.00	NS
*B. pertussis*										1.00

*S. pneumoniae, Streptococcus pneumoniae; RSV, respiratory syncytial virus; RV, rhinovirus; PIV, parainfluenza virus; NS, nonsignificant. Correlations >0.50 in **boldface**; p value >0.05. †Unlagged.

### Linear Regression Models

Table 4 presents, for each registry, the time lag (in weeks) that maximized the model fit of regressing syndrome on pathogens. For the GP, hospital, mortality, and pharmacy data, the respiratory pathogens explained the syndrome variation very well (78%–86%). Variations in the absenteeism syndrome could be explained for 68% by variations in the pathogen counts. Although the laboratory submissions syndrome had the lowest explained variance, still 61% of the variations in this syndrome were explained by variations in pathogen counts. Hierarchical time-series models did not show significant autocorrelation in the residuals of the models with pathogen counts as explanatory variables (*20*,*21*).

**Table 4:** All respiratory pathogen counts included as explanatory variables in the regression models, the Netherlands, 1999–2004*†

Syndrome data	RSV	Influenza A	Influenza B	*S. pneumoniae* (residual)	RV	PIV	Adenovirus	Enterovirus	*Mycoplasma pneumoniae*	*Bordetella pertussis*
Absenteeism	2	5	4	2	4	5	–	–	–	­
GP	–1	1	2	–1	1	2	−2	–	–3	­
Pharmacy	–1	0	2	0	2	5	−2	–	5	−3
Hospitalization	0	2	1	–	–2	3	–	–	–	­
Laboratory submissions	–2	0	1	–3	–	2		–	5	­
Mortality	−3	1	0	–	–	–	–	–	–	­

*S. pneumoniae, Streptococcus pneumoniae; RSV, respiratory syncytial virus; RV, rhinovirus; PIV, parainfluenza virus; GP, general practice; –, pathogen not included in model. †The lag time (in weeks) is indicated, that showed optimal fit between syndrome time-series and lagged pathogen counts included in the linear regression model; e.g., according to the model, the trend in hospitalizations precedes the influenza A laboratory counts by 2 weeks.

When seasonal terms were added to the model, the variations in the mortality syndrome were just as well explained as by the model with only pathogen counts (Table 5; R^2^ remains 78%), while by the model with only seasonal terms, the explained variance was much lower (only 52%, Table 5). For the hospitalizations, laboratory submissions, and GP data, only slightly more syndrome variation was explained by adding seasonal terms. With only seasonal terms, the explained variance for these syndromes was clearly lower than with only pathogens in the models (8%–11% lower, Table 5). However, for the absenteeism and, to a lesser extent, the pharmacy data, the model with both pathogen and seasonal terms clearly explained more syndrome variations (Table 5, absenteeism 68% vs. 80%; pharmacy 80% vs. 87%). Furthermore, for the absenteeism data, the model with only seasonal terms had an even higher R^2^ than the model with only pathogens, whereas for the pharmacy data, the R^2^ with only seasonal terms was only slightly lower (3%, Table 5).

**Table 5:** Syndrome variation that can be explained by either the pathogen counts,  seasonal terms, or pathogen counts and seasonal terms together*

Syndrome data	Pathogens, %	Pathogens and seasonal terms, %	Seasonal terms, %
Absenteeism	68	80	79
GP	86	89	75
Pharmacy	80	87	77
Hospitalization	84	88	75
Laboratory submissions	61	63	53
Mortality	78	78	52

*Estimated by 3 different R^2^ values for each registry: 1) for the syndromes explained by pathogen counts alone; 2) after adding seasonal terms to the pathogen model; and 3) for the syndromes explained by seasonal terms alone (sine and cosine parameters). GP, general practice.

Table 6 shows that for mortality, hospitalizations, laboratory submissions, and GP data, the pathogens with the highest effect clearly were RSV, influenza A, and influenza B, with no or only modest decline in standardized parameter estimates after adding seasonal terms. For the GP and hospital data, some pathogens became insignificant after seasonal terms were added (GP: rhinovirus and adenovirus; hospital: parainfluenza virus). For the pharmacy data, half of all pathogen variables became insignificant after seasonal terms were added, whereas for the absenteeism data, almost all pathogens became insignificant (Table 6).

**Table 6:** Standardized parameter estimates (βs) for all respiratory pathogen counts included as explanatory variables in the regression models: before and after adding seasonal terms to the models*†

Syndrome data	RSV	Influenza A	Influenza B	*S. pneumoniae* (residual)	RV	PIV	Adenovirus	Enterovirus	*Mycoplasma pneumoniae*	*Bordetella pertussis*
Absenteeism	0.31/ (NS)	0.27/ (NS)	0.33/ (NS)	0.28/ 0.12	0.19/ (NS)	0.20/ (NS)	–	_	_	_
GP	0.60/ 0.51	0.32/ 0.32	0.20/ 0.16	0.13/ 0.10	0.07/ (NS)	0.14/ 0.08	0.07/ (NS)	_	0.06/ 0.05	_
Pharmacy	0.51/ 0.54	0.27/ 0.22	0.24/ (NS)	0.25/ 0.11	0.16/ 0.08	0.16/ (NS)	0.08/ (NS)	_	0.12/ (NS)	0.11/ 0.11
Hospitalization	0.60/ 0.44	0.36/ 0.34	0.21/ 0.12	_	0.13/ 0.05	0.09/ (NS)	_	_	_	_
Laboratory submissions	0.49/ 0.47	0.19/ 0.20	0.22/ 0.18	0.28/ 0.22	_	0.17/ 0.08	_	_	0.10/ 0.10	_
Mortality	0.40/ 0.36	0.52/ 0.51	0.24/ 0.24	_	_	_	_	_	_	_

*S. pneumoniae, Streptococcus pneumoniae; RSV, respiratory syncytial virus; RV, rhinovirus; PIV, parainfluenza virus; GP, general practice; –, pathogen not included in model; NS, the pathogen variable is no longer significant after seasonal terms are added. †For example, 0.60/0.40 for RSV indicates a standardized β of 0.60 for RSV in the model with only pathogen variables and a β of 0.40 in the same model after adding seasonal terms.

Several syndrome observations exceeded the 95% UCLs of the models (0–10/registry/year), which indicates that those syndrome observations deviated strongly from model predictions. The recurrent elevation in October of the absenteeism, GP, and pharmacy syndrome several times exceeded the UCLs (October 2001: pharmacy and GP; 2002: absenteeism; 2003: GP, absenteeism; not shown), which indicated that the model could not explain these elevations.

### Timeliness

**Figure 2:**
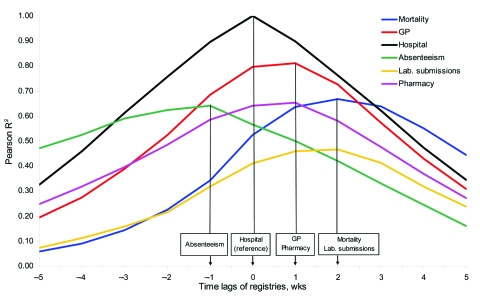
The (maximum) R^2^ by the lagged syndromes with the hospital syndrome as a reference. Aggregated by week, univariate Pearson correlation coefficients were calculated of the hospital syndrome and each of the other syndromes. Note that the Pearson correlation coefficients are calculated over different periods for the different registries because not all registries cover the same period (Table 1). Measured by the syndrome lag with the maximized R^2^, the timeliness differed between the registries in the following order: absenteeism, hospital, pharmacy/general practice (GP), mortality/laboratory submissions (as projected on the x-axis).

In Figure 2, for each registry, the difference in timeliness with the hospital registry is indicated by the lag that maximizes R^2^. The absenteeism syndrome (green line) preceded the hospital syndrome by 1 week, followed by the GP-based and prescription-based syndromes at +1 week and the syndrome based on mortality and laboratory submission data at +2 weeks after the hospital syndrome (projected on x-axis, Figure 2).

The differences in timeliness between the syndromes and the pathogen surveillance data were reflected by the regression models relating the syndromes to the (positive or negative) lagged pathogens (Table 4). Influenza A and influenza B had lags of 0–5 weeks, which suggests that the registry-syndromes were 0–5 weeks ahead of laboratory counts for these infections. Fluctuations in the time series of respiratory hospitalizations and the laboratory RSV counts seemed to appear in the same week (lag = 0). All other syndromes appeared to be 1–3 weeks later than the RSV counts, except absenteeism, which is 2 weeks earlier. Again, absenteeism seemed to be the earliest syndrome (2–5 weeks earlier than RSV, influenza A, and influenza B), followed by the hospital syndrome (0–2 weeks earlier), the GP-based and prescription-based syndromes (2 weeks earlier until 1 week later), the laboratory submission syndrome (1 week earlier until 2 weeks later), and the mortality syndrome (0–3 weeks later than RSV, influenza A, and influenza B).

## Discussion

We explored the potential of 6 Dutch medical registries for respiratory syndromic surveillance. Although several other studies also evaluated routine (medical) data for syndromic surveillance purposes (*22*–*27*), most evaluated only 1 syndrome and correlated this only to influenza data. An exception is Bourgeois et al. (*24*), who validated a respiratory syndrome in relation to diagnoses of several respiratory pathogens in a pediatric population, and Cooper et al. (*27*), who estimated the contribution of specific respiratory pathogens to variations in respiratory syndromes. Both studies concluded that RSV and influenza explain most of the variations in these syndromes, consistent with our findings.

Our study shows that all syndrome data described in this study showed higher levels in winter, which corresponded to the seasonal patterns of RSV, *S. pneumoniae*, and influenza A and B viruses. Linear regression showed that the syndromes can be explained by lagged laboratory counts for respiratory pathogens (up to 86%, highest effect of influenza A, influenza B, and RSV), which indicates their potential usefulness for syndromic surveillance. Timeliness differed, with up to 5 weeks potential gain in early warning by syndromic data, compared with routine laboratory surveillance data.

A limitation of our study is the short duration of our time series, especially for absenteeism and pharmacy data. Therefore, whether our observed associations between syndromes and pathogen counts can be generalized remains unclear.

We relied on laboratory pathogen counts as a proxy for their prevalence and the illness they cause. Changes in test volume over time would result in misclassification bias (as noncausative pathogens will be detected as well). However, such changes are presumably dwarfed by changes during “truly” epidemic elevations of common respiratory pathogens. Additionally, laboratory diagnostics are mostly performed on hospitalized patients, and thus results inadequately reflect activity of pathogens that predominantly cause mild illness.

By adding seasonal terms, we observed that for the absenteeism and, to a lesser extent, the pharmacy registry, the associations between the respiratory syndromes and the pathogen counts might be biased to some extent. For the GP, hospital, laboratory submission, and mortality data, season is probably not an important confounder for the association between the syndromes and pathogens, because including seasonal terms in the models resulted in the same or only slightly higher explained syndrome variance (measured by R^2^). Models with seasonal terms alone mostly had lower explained variance than the pathogen models. For the GP and hospital data, some pathogens became insignificant after seasonal terms were added (Table 6) but not those pathogens with the largest effect estimates (RSV, influenza A and B). Therefore, we are confident in concluding that the GP, hospital, laboratory submission, and mortality syndromes do reflect pathogen activity sufficiently for use in syndromic surveillance.

The higher R^2^ value of the absenteeism model with seasonal terms alone suggests seasonality of absenteeism caused by several nonrespiratory conditions (*28*,*29*). To some extent, this also applies to the pharmacy syndrome, which includes medications that are not specific for respiratory infections (e.g., antimicrobial drugs). This could be validated in future studies by linking medications to illness. However, for both the absenteeism and pharmacy syndromes, the variation explained by seasonal terms is probably overestimated to some extent because data for only 2 and 3 years were used. Consequently, these time series contained less information on variation between different years than for the other registries, which benefits fitting of a model with several sine and cosine terms.

To our knowledge, laboratory submission data (test requests) have not been evaluated before as a data source for syndromic surveillance. The modest explained variance for the laboratory submissions syndrome could possibly reflect the limited use in our country of laboratory testing algorithms, which leads to substantial differences in diagnostic regimes for patients with similar clinical symptoms. In addition, occasional extra alertness by clinicians can make these data unreliable for surveillance. For instance, an unusual peak was observed in the laboratory submissions syndrome in 1999, after the official announcement of an outbreak of Legionnaires’ disease (*30*).

An unexpected increase was also observed in the absenteeism, GP, and pharmacy syndromes, which occurred consistently each year around October (2001–2004). These peaks preceded the syndrome peaks concurring with peaks in influenza A, influenza B, and RSV counts and may be caused by rhinovirus activity—and asthma exacerbations caused by rhinovirus—which usually rises in the fall (*31*–*33*). Rhinovirus might go undetected because GP physicians rarely ask for diagnostics if they suspect a nonbacterial cause for relatively mild respiratory disease. Although specific asthma diagnoses were excluded from the respiratory syndrome definitions, exacerbations of asthma might affect other respiratory categories in the GP or pharmacy syndrome. This observation illustrates that additional diagnostics are needed for identifying the causes of unexplained respiratory disease elevations. Several novel respiratory pathogens for which diagnostics are not yet widely available have been discovered in recent years, underlining that it is quite possible that “hidden” epidemics occur (*34*–*36*). The extra October peak and several other syndrome elevations above the 95% UCLs in our study may well reflect such hidden epidemics. The fact that these occur is supported by studies showing that many individual syndrome cases cannot be linked to known pathogens. For example, Cooper et al. (*37*), who investigated syndromic signals by using patient self-sampling (at home), could only obtain diagnostic results for 22% of these cases.

For early warning surveillance, timeliness is crucial. Absenteeism data seem to have the best timeliness, but their lack of medical detail complicates interpretation. Unexpectedly, the hospital data reflect respiratory pathogen activity earlier than the GP data. Although in the Netherlands patients are encouraged to consult their GP before going to the hospital, elderly persons, for whom respiratory infections are more likely to cause severe illness, may often go to a hospital directly. Therefore, hospital data may prove to be an earlier marker for respiratory disease than GP data, but this possibility needs further exploration.

An important concern when using syndromic surveillance is that it may generate nonspecific alerts, which, if they happen regularly, would lead to lack of confidence in a syndrome-based surveillance system. Here, we see a clear advantage of using data from multiple registries in parallel so that signal detection can be made more specific by focusing on signals that occur concurrently in >1 data source. To illustrate this we defined every exceeding of the UCLs of the regression models as a “signal,” i.e., a syndrome elevation unexplained by known pathogen activity and therefore possibly reflecting activity of underdiagnosed or emerging infectious disease. Over 2002–2003 (the period that all 6 registries were in the study), only 5 “concurrent” signals occurred versus 34 “single” signals over all registries. We did not evaluate whether the syndromes indeed detect outbreaks of infectious diseases earlier than clinical or laboratory pathogen surveillance. Such an evaluation is often performed by testing the ability to detect historical natural outbreaks or simulated outbreaks (*10*,*38*). However, historical natural outbreaks are rare and simulated outbreaks may be unrealistic. Nevertheless, further research into the outbreak detection performance of these syndromes would be worthwhile.

The results of this study suggest that it might be best to combine syndromic data and pathogen counts in a prospective surveillance system. Such surveillance can identify distinct syndrome elevations that cannot be explained by respiratory pathogen activity as indicated by routine laboratory pathogen surveillance.

## Conclusion

Overall, the GP, hospital, mortality and, to a lesser extent, laboratory submission syndromes reflect week-to-week fluctuations in the time-series of respiratory pathogens as detected in the laboratory. Registries monitoring trends of these syndromes will therefore most likely reflect illness caused by emerging or underdiagnosed respiratory pathogens as well and therefore are suited for syndromic surveillance. Further research would be required to assess to what extent absenteeism and pharmacy data reflect respiratory illness. Investigating the actual outbreak detection performance of the syndromes in this study would also be worthwhile.

Data from the registries in this study are not yet real-time available, although given modern information technology, this availability is clearly feasible. Our study can help prioritize which type of healthcare data to include in future syndromic real-time surveillance systems.

## Supplementary Material

Technical Appendix Validation of Syndromic Surveillance for Respiratory Pathogen Activity
