# Supplementary material for: Validation of Syndromic Surveillance for Respiratory Pathogen Activity
Source: Emerg Infect Dis. 2008 Jun;14(6):917–25. doi: 10.3201/eid1406.071467 (PMC2600280; doi:10.3201/eid1406.071467)
Supplement: Technical Appendix — Validation of Syndromic Surveillance for Respiratory Pathogen Activity [file 07-1467_Techapp-s1.pdf]

Validation of Syndromic Surveillance for Respiratory Pathogen Activity

Technical Appendix

Detailed Syndrome Definitions for Each Syndrome Data Source

A general respiratory syndrome was defined for each data source (except for the absenteeism data, which contain no medical information; see Table 1). We used the International Classification of Diseases, 9th revision, Clinical Modification (ICD-9-CM*)* codes as selected by the Centers for Disease Control and Prevention (CDC), Atlanta, Georgia, USA (www.bt.cdc.gov/surveillance/syndromedef). To define a respiratory syndrome, we selected both the codes for general respiratory symptoms and diagnoses (category 1 in CDC list) and the codes for specific respiratory biologic agent diagnoses (category 3 in CDC list). For the hospital data (see Table 1), we used these syndrome codes with some minor adaptations for the Dutch version of ICD-9-CM. For the mortality data (see Table 2) the ICD-9-CM codes were converted into ICD 10th revision (ICD-10) codes by using the World Health Organization ICD-9/ICD-10 translation list and expert opinion, if necessary (ICD-9/ICD-10 Translator; see www.who.int/classifications/en). For the GP consultation data (see Table 3), International Classification of Primary Care (ICPC) codes were included in a respiratory syndrome by expert opinion, guided by the CDC respiratory syndrome case definition.

For a respiratory syndrome definition based on the pharmacy data, we used Anatomical Therapeutic Chemical Classification System (ATC) codes of medications that experts considered indicative for respiratory infectious disease complaints. Of those, we included only ATC-5 codes that had higher levels in winter. See Table 4 for the specific included ATC-5 codes.

For a respiratory syndrome definition based on the laboratory submissions data, we included all submissions for specific diagnostics that are known to be of respiratory cause: 1) all submissions for microbiologic diagnostic tests on respiratory materials (sputum, bronchoalveolar lavage, pleural liquid); 2) all submissions for serology on known specific respiratory pathogens

(see list of serologic tests in Table 5); 3) all submissions for *Legionella* spp. or *Streptococcus pneumoniae* antigen tests on urine.

For all data types we assumed that in a prospective setting real-time syndrome-classification would be feasible (on date of consultation/hospitalization/death/submission/ dispense).

Table 1. ICD-9-CM codes for the respiratory syndrome in hospital data*

ICD-9-CM code	Description
020.3	Primary pneumonic plague
020.4	Secondary pneumonic plague
020.5	Pneumonic plague not otherwise specified
021.2	Pulmonary tularemia
022.1	Pulmonary anthrax
031.0	Mycobacteria, pulmonary
031.8	Other specified mycobacterial diseases
031.9	Mycobacteria diseases/unspecified
032.0	Faucial diphtheria
032.1	Nasopharynx diphtheria
032.2	Anterior nasal diphtheria
032.3	Laryngeal diphtheria
032.89	Diphtheria not elsewhere classified
032.9	Diphtheria not otherwise specified
033.0	*Bordetella pertussis*
033.1	*Bordetella parapertussis*
033.8	Whooping cough not elsewhere classified
033.9	Whooping cough (unspecified organism)
034.0	Streptococcal sore throat
055.1	Postmeasles pneumonia
055.2	Postmeasles otitis media
073.0	Ornithosis, with pneumonia
073.7	Ornithosis, with other specified complication
073.8	Ornithosis, with unspecified complication
073.9	Ornithosis, unspecified
079.0	Adenovirus infection not otherwise specified
079.1	Echovirus infection not otherwise specified nos.
079.2	Coxsackie virus
079.3	Rhinovirus infection not otherwise specified
079.8	Viral infection in conditions classified elsewhere and of unspecified site
098.6	Gonoccocal, infection of pharynx
114.0	Primary coccidioidomycosis (lung)

114.5	Pulmonary coccidioidomycosis, unspecified
114.9	Coccidioidomycosis not otherwise specified
115.00	Histoplasmosis, without mention of manifestation
115.05	*Histoplasma capsulatum* pneumonia
115.09	*Histoplasma capsulatum* not elsewhere classified
115.10	*Histoplasma duboisii* not otherwise specified
115.15	*Histoplasma duboisii* pneumonia
115.90	Histoplasmosis, without manifestation
115.95	Histoplasmosis pneumonia
115.99	Histoplasmosis not elsewhere classified
116.0	Blastomycosis
116.1	Paracoccidioidomycosis
117.1	Sporotrichosis
117.3	Pulmonary aspergillosis
117.5	Cryptococcosis
130.4	*Toxoplasma* pneumonitis
136.3	Pneumocystosis
460	Nasopharyngitis, acute
462	Pharyngitis, acute not otherwise specified
463	Tonsillitis, acute
464.0	Acute laryngitis
464.10	Tracheitis without obstruction
464.11	Acute tracheitis with obstruction
464.20	Laryngotracheitis without obstruction
464.21	Acute laryngotracheitis with obstruction
464.30	Epiglottitis acute without obstruction
464.31	Acute epiglottitis with obstruction
464.4	Croup
465.0	Laryngopharyngitis, acute
465.8	Upper respiratory infection, other multiple sites
465.9	Upper respiratory infection, acute not otherwise specified
466.0	Bronchitis acute
466.1	Acute bronchiolitis
478.9	Respiratory tract disease
480.0	Adenoviral pneumonia
480.1	Pneumonia due to respiratory syncytial virus
480.2	Parinfluenza viral pneumonia
480.8	Viral pneumonia not elsewhere classified
480.9	Pneumonia, viral
481	Pneumococcal pneumonia (lobar)
482.0	Pneumonia due to *Klebsiella pneumoniae*
482.1	Pneumonia due to *Pseudomonas*
482.2	*Haemophilus influenzae* pneumonia
482.3	Pneumonia due to *Streptococcus*

482.4	Pneumonia due to *Staphylococcus*
482.8	Pneumonia due to bacteria not elsewhere classified
482.9	Pneumonia due to bacteria not otherwise specified
483	Pneumonia due to organism not elsewhere classified
484.1	Pneumonia due to cytomegalic inclusion disease
484.3	Pneumonia in whooping cough
484.5	Pneumonia in anthrax
484.6	Pneumonia in aspergillosis
484.7	Pneumonia in other systemic mycoses
484.8	Pneumonia in infection disease not elsewhere classified
485	Bronchopneumonia organism unspecified
486	Pneumonia, organism not otherwise specified
487.0	Influenza with pneumonia
487.1	Influenza with other respiratory manifestations
487.8	Influenza with other manifestations
490	Bronchitis not otherwise specified
511.0	Pleurisy without mention of effusion or current tuberculosis
511.1	Pleurisy with effusion, with mention of a bacterial cause other than tuberculosis
511.8	Hemothorax
513.0	Abscess lung
513.1	Abscess of mediastinum
518.4	Edema lung acute not otherwise specified
518.8	Other diseases of lung not otherwise classified
519.2	Mediastinitis
519.3	Mediastinum, diseases not elsewhere classified
769	Respiratory distress syndrome
786.00	Respiratory abnormality
786.09	Other specified respiratory abnormality
786.1	Stridor
786.2	Cough
786.3	Hemoptysis
786.52	Painful respiration/pleurodynia
799.1	Respiratory arrest

*ICD-9-CM, International Classification of Diseases, 9th Revision, Clinical Modification.

Table 2. ICD-10 codes for the respiratory syndrome in mortality data*

ICD-10 code	Description
A202	Pneumonic plague
A212	Pulmonary tularemia
A221	Pulmonary anthrax
A310	Pulmonary mycobacterial infection
A318	Other mycobacterial infections
A319	Mycobacterial infection, unspecified
A360	Pharyngeal diphtheria

A361	Nasopharyngeal diphtheria
A362	Laryngeal diphtheria
A368	Other diphtheria
A369	Diphtheria, unspecified
A370	Whooping cough due to *Bordetella pertussis*
A371	Whooping cough due to *Bordetella parapertussis*
A378	Whooping cough due to other *Bordetella* species
A379	Whooping cough, unspecified
A481	Legionnaires' disease
A545	Gonococcal pharyngitis
A70	*Chlamydia psittaci* infection
B012	Varicella pneumonia (J17.1*)
B052	Measles complicated by pneumonia (J17.1*)
B053	Measles complicated by otitis media (H67.1*)
B340	Adenovirus infection, unspecified
B341	Enterovirus infection, unspecified
B342	Coronavirus infection, unspecified
B348	Other viral infections of unspecified site
B380	Acute pulmonary coccidioidomycosis
B382	Pulmonary coccidioidomycosis, unspecified
B389	Coccidioidomycosis, unspecified
B390	Acute pulmonary histoplasmosis capsulatum
B392	Pulmonary histoplasmosis capsulatum, unspecified
B393	Disseminated histoplasmosis capsulatum
B394	Histoplasmosis capsulatum, unspecified
B395	Histoplasmosis duboisii
B399	Histoplasmosis, unspecified
B400	Acute pulmonary blastomycosis
B402	Pulmonary blastomycosis, unspecified
B407	Disseminated blastomycosis
B408	Other forms of blastomycosis
B409	Blastomycosis, unspecified
B410	Pulmonary paracoccidioidomycosis
B417	Disseminated paracoccidioidomycosis
B418	Other forms of paracoccidioidomycosis
B419	Paracoccidioidomycosis, unspecified
B420	Pulmonary sporotrichosis (J99.8*)
B427	Disseminated sporotrichosis
B428	Other forms of sporotrichosis
B429	Sporotrichosis, unspecified
B440	Invasive pulmonary aspergillosis
B441	Other pulmonary aspergillosis
B442	Tonsillar aspergillosis
B447	Disseminated aspergillosis

B448	Other forms of aspergillosis
B449	Aspergillosis, unspecified
B450	Pulmonary cryptococcosis
B457	Disseminated cryptococcosis
B458	Other forms of cryptococcosis
B459	Cryptococcosis, unspecified
B583	Pulmonary toxoplasmosis (J17.3*)
B59	Pneumocystosis
B970	Adenovirus as the cause of diseases classified to other chapters
B971	Enterovirus as the cause of diseases classified to other chapters
B972	Coronavirus as the cause of diseases classified to other chapters
B974	Respiratory syncytial virus as the cause of diseases classified to other chapters
B978	Other viral agents as the cause of diseases classified to other chapters
G473	Sleep apnea
J00	Acute nasopharyngitis (common cold)
J020	Streptococcal pharyngitis
J028	Acute pharyngitis due to other specified organisms
J029	Acute pharyngitis, unspecified
J030	Streptococcal tonsillitis
J038	Acute tonsillitis due to other specified organisms
J039	Acute tonsillitis, unspecified
J040	Acute laryngitis
J041	Acute tracheitis
J042	Acute laryngotracheitis
J050	Acute obstructive laryngitis (croup)
J051	Acute epiglottitis
J060	Acute laryngopharyngitis
J068	Other acute upper respiratory infections of multiple sites
J069	Acute upper respiratory infection, unspecified
J100	Influenza with pneumonia, influenza virus identified
J101	Influenza with other respiratory manifestations, influenza virus identified
J108	Influenza with other manifestations, influenza virus identified
J110	Influenza with pneumonia, virus not identified
J111	Influenza with other respiratory manifestations, virus not identified
J118	Influenza with other manifestations, virus not identified
J120	Adenoviral pneumonia
J121	Respiratory syncytial virus pneumonia
J122	Parainfluenza virus pneumonia
J128	Other viral pneumonia
J129	Viral pneumonia, unspecified
J13	Pneumonia due to *Streptococcus pneumoniae*
J14	Pneumonia due to *Haemophilus influenzae*
J150	Pneumonia due to *Klebsiella pneumoniae*
J151	Pneumonia due to *Pseudomonas*

J152	Pneumonia due to *Staphylococcus*
J153	Pneumonia due to *Streptococcus*, group B
J154	Pneumonia due to other streptococci
J155	Pneumonia due to *Escherichia coli*
J156	Pneumonia due to other aerobic Gram-negative bacteria
J157	Pneumonia due to *Mycoplasma pneumoniae*
J158	Other bacterial pneumonia
J159	Bacterial pneumonia, unspecified
J160	Chlamydial pneumonia
J168	Pneumonia due to other specified infectious organisms
J170	Pneumonia in bacterial diseases classified elsewhere
J171	Pneumonia in viral diseases classified elsewhere
J172	Pneumonia in mycoses
J173	Pneumonia in parasitic diseases
J178	Pneumonia in other diseases classified elsewhere
J180	Bronchopneumonia, unspecified
J182	Hypostatic pneumonia, unspecified
J188	Other pneumonia, organism unspecified
J189	Pneumonia, unspecified
J200	Acute bronchitis due to *Mycoplasma pneumoniae*
J201	Acute bronchitis due to *Haemophilus influenzae*
J202	Acute bronchitis due to streptococcus
J203	Acute bronchitis due to coxsackievirus
J204	Acute bronchitis due to parainfluenza virus
J205	Acute bronchitis due to respiratory syncytial virus
J206	Acute bronchitis due to rhinovirus
J207	Acute bronchitis due to echovirus
J208	Acute bronchitis due to other specified organisms
J209	Acute bronchitis, unspecified
J210	Acute bronchiolitis due to respiratory syncytial virus
J218	Acute bronchiolitis due to other specified organisms
J219	Acute bronchiolitis, unspecified
J22	Unspecified acute lower respiratory infection
J398	Other specified diseases of upper respiratory tract
J40	Bronchitis, not specified as acute or chronic
J850	Gangrene and necrosis of lung
J851	Abscess of lung with pneumonia
J852	Abscess of lung without pneumonia
J853	Abscess of mediastinum
J942	Hemothorax
J949	Pleural condition, unspecified
J960	Acute respiratory failure
J969	Respiratory failure, unspecified
J985	Diseases of mediastinum, not elsewhere classified

J998	Respiratory disorders in other diseases classified elsewhere
P220	Respiratory distress syndrome of newborn
R042	Hemoptysis
R049	Hemorrhage from respiratory passages, unspecified
R05	Cough
R061	Stridor
R063	Periodic breathing
R064	Hyperventilation
R065	Mouth breathing
R068	Other and unspecified abnormalities of breathing
R071	Chest pain on breathing
R091	Pleurisy
R092	Respiratory arrest

*ICD-10, International Classification of Diseases, 10th Revision.

Table 3. ICPC codes for the respiratory syndrome in general practice consultations data*

ICPC codes	Description
H71	Acute otitis media/myringitis
L04	Chest symptom/complaint
R01	Pain respiratory system
R02	Shortness of breath/dyspnea
R03	Wheezing
R04	Breathing problem, other
R05	Cough
R07	Sneezing/nasal congestion
R21	Throat symptom/complaint
R24	Hemoptysis
R29	Respiratory symptom/complaint, other
R71	Whooping cough
R74	Upper respiratory infection, acute
R75	Sinusitis acute/chronic
R76	Tonsillitis, acute
R77	Laryngitis/tracheitis acute
R78	Acute bronchitis/bronchiolitis
R80	Influenza
R81	Pneumonia
R82	Pleurisy/pleural effusion
R83	Respiratory infection, other
R93	Pleural effusion not otherwise specified
R99	Respiratory disease, other

*ICPC, International Classification of Primary Care.

Table 4. ATC level 5 codes for the respiratory syndrome in pharmacy data*

ATC-5 code	Description
J01AA	Tetracyclines
J01CA	Penicillins with extended spectrum
J01CR	Combinations of penicillins, including β-lactamase inhibitors
J01FA	Macrolides
R05CA	Expectorants
R05DA	Opium alkaloids and derivatives
R06AD	Phenothiazine derivatives

*ATC, Anatomical Therapeutic Chemical Classification System.

Table 5. Serologic test subjects included in the respiratory syndrome for laboratory submissions (see information on other included tests in text)

Serologic tests performed on
Adenovirus 2
Adenovirus
Antibodies to adenovirus
Antibodies to *Aspergillus fumigatus*
Antibodies to *Aspergillus* species
Antibodies to *Chlamydia pneumoniae*
Antibodies to *Chlamydia psittaci*
Antibodies to *Chlamydia* species
Antibodies to coronavirus
Antibodies to *Corynebacterium diphtheriae*
Antibodies to influenza A virus
Antibodies to influenza B virus
Antibodies to *Legionella*
Antibodies to *Legionella pneumophila*
Antibodies to *Legionella pneumophila* serogroup 1
Antibodies to *Mycoplasma pneumoniae*
Antibodies to parainfluenza 1 virus
Antibodies to parainfluenza 2 virus
Antibodies to parainfluenza 3 virus
Antibodies to parainfluenza virus
Antibodies to respiratory syncytial virus
Antibodies to *Streptococcus pneumoniae*
Antigen *Aspergillus fumigatus*
Antigen *Aspergillus* species
IgA *Chlamydia pneumoniae*
IgA *Chlamydia* species
IgA *Mycoplasma pneumoniae*
IgG adenovirus
IgG *Leptospira*
IgG *Aspergillus fumigatus*
IgG *Chlamydia pneumoniae*
IgG *Chlamydia psittaci*
IgG *Chlamydia* species
IgG influenza virus A

IgG influenza virus B
IgG *Legionella pneumophila*
IgG *Legionella* species
IgG *Mycoplasma pneumoniae*
IgG parainfluenza 1 virus
IgG parainfluenza 2 virus
IgG parainfluenza 3 virus
IgG respiratory syncytial virus
IgG *Streptococcus pneumoniae*
IgM influenza virus A
IgM *Chlamydia psittaci*
IgM *Chlamydia* species
IgM influenza B virus
IgM *Legionella pneumophila*
IgM *Legionella* species
IgM *Mycoplasma pneumoniae*
IgM *Mycoplasma* species
IgM parainfluenza 1 virus
IgM parainfluenza 2 virus
IgM parainfluenza 3 virus

*IG, immunoglobulin.

Details on the Regression Model Variables

We constructed a multiple linear regression model:

$S_t = b_0 + b_1 P_{A,t+x} + b_2 P_{B,t+y} + \ldots + R_{,t}$

S = level of a respiratory syndrome

t = time in weeks

$P_{A/B/etc}$ = lagged respiratory pathogens detected in the laboratory

x/y/etc = lag time in weeks, for shifting the pathogen time series over a range of -5 up to +5 weeks.

R = residual of the model

A forward stepwise regression approach was used, each step selecting the lagged pathogen that contributed most to the model fit (assessed with Akaike's information criterion). Each pathogen was included in the model only once and only if it contributed significantly ($p<0.05$). Negative associations were excluded to avoid biologically implausible associations in the models between

the pathogens and the syndromes (e.g., negative associations between enteroviruses, which peak in summer, and respiratory syndromes, which peak in winter). We checked for significant autocorrelation in the residual of the models.

To investigate whether seasonal variation could be a confounder for the association between pathogens and syndromes we then calculated 3 R^2 values for the models: 1) with only pathogen variables, 2) after adding seasonal terms (sine(k2πweek/52) and cosine(k2πweek/52), k = 1, 2, 3), and 3) with only seasonal terms. We calculated the standardized parameter estimates as well, before and after adding seasonal terms. The standardized parameter estimates are the beta values that result when all variables are standardized to a mean of 0 and a variance of 1. These estimates are computed by multiplying the original estimates by the standard deviation of the regressor (independent) variable and then dividing by the standard deviation of the dependent variable.
